# Detection of colon polyps by a novel, polymer pattern-based full blood test

**DOI:** 10.1186/1479-5876-11-278

**Published:** 2013-11-04

**Authors:** Markus Franz, Matthias Scholz, Ilka Henze, Stefan Röckl, Luis I Gomez

**Affiliations:** 1 Institute of Clinical Immunology, Medical Faculty, Universität Leipzig, Johannisallee 30, Leipzig D-04103, Germany; 2 Kapelan Bio-Imaging GmbH, Prager St 60, Leipzig D-04317, Germany; 3 Private practice, Röntgenst 6-8, Langen D-63225, Germany; 4 Translational Centre for Regenerative Medicine (TRM), Universität Leipzig, Philipp-Rosenthal-St 55, Leipzig D-04103, Germany; 5 Private practice, Schulst 1, Cham D-93413, Germany; 6 INDAGO GmbH, Deutscher Platz 5a, Leipzig D-04103, Germany

**Keywords:** Colon polyps, Colorectal cancer, Blood-test, Polyp specific polymer analysis, Polymeric patterns

## Abstract

Numerous studies have shown that early screening for the presence of pre-cancerous colon polyps and their subsequent removal decreases the risk of developing colon cancer. Colonoscopy is currently the most effective screening method, but due to the invasive nature of the procedure many patients avoid forgo testing. Futhermore, the procedure itself requires perfect execution by the gastroenterologist. Against this backdrop, a non-invasive blood screening method for the detection of colon polyps that has higher sensitivity than current screening techniques would be beneficial in the early identification of patients at risk for colon cancer. A prospective, double-blinded, controlled clinical study was designed to demonstrate the diagnostic performance of Polyp Specific Polymer analysis, a novel laboratory methodology. The primary objective of this clinical trial was to estimate the diagnostic accuracy of the Polyp Specific Polymer analysis for colon polyps using colonoscopy and histological tests as the diagnostic accuracy standards. Secondary objectives of this trial included estimating positive and negative predictive values for colon polyps, investigating reliability, determining covariates influencing diagnostic accuracy and obtaining absolute and relative frequencies of valid test results.

In patients undergoing screening colonoscopy and histology examination, a sensitivity of 72.4% and a specificity of 62.3% could be proven.

These results indicate that using this improved screening method it is possible to effectively identify the highest-risk candidates for endoscopy, thereby advancing the goal of decreasing the incidence or mortality of colorectal cancer in the selected population. Moreover, this diagnostic tool has potential socio-economic implications, conserving healthcare resources by enabling higher patient selectivity for endoscopy and eventual transfer to curative prevention via polypectomy.

By combining the best-established low-risk screening elements together with a validated, highly sensitive blood test as described in this study, a steadfast increase in the estimation of colorectal cancer-risk before colonoscopy can be expected.

## Background

Colon polyps are mucosal lesions that protrude into the lumen of the large intestine and are highly prevalent in Western populations in people over 55 years of age [1]. There are four types of colonic polyps: the most important are adenomatous polyps, which are implied as precursors for colorectal cancer (CRC), the second leading cause of cancer related deaths in Western countries. One of the most insidious problems regarding colon polyps is that the majority of affected persons do not actually display any adverse symptoms. Numerous studies have indicated that early screening for the presence of pre-cancerous colon polyps and their subsequent removal decreases the probability of colon cancer and associated mortality [2,3].

There are various diagnostic methods available to detect colonic polyps. These include: fecal occult blood testing (FOB), virtual colonoscopy, sigmoidoscopy, colonoscopy, and the combination of barium enema and sigmoidoscopy. An estimated 1-5% of patients over 55 years of age have a positive fecal occult blood test, approximately 2-10% of these have cancer while 20-30% have adenomas or polyps [1,4]. Colonoscopy, which allows the physician to unambiguously identify colon polyps and remove the polyps for analysis, is the most effective screening method [2]. However, because of the invasive nature of the procedure, which must be performed by qualified physicians in a clinical setting, many patients that would potentially benefit from the procedure abstain from testing.

The progression of adenoma to carcinoma represents a significant public health problem and early screening for and removal of colon polyps has been demonstrated to reduce the risk of death from colorectal cancer [4]. A non-invasive blood or serum-based screening method for the detection of colon polyps that has a higher sensitivity and specificity than current screening techniques and that could be carried out with conventional blood tests would be beneficial in early identification of patients at risk for colon cancer [5].

Polyp Specific Polymer (PSP) analysis is a novel laboratory methodology for the diagnosis of human diseases that can detect various pathological changes in a variety of organs and organ systems [3]. PSP analysis detects aberrant composition of whole blood providing information about organ function and their disorders. As yet, such broad information could not be detected or could only be detected with considerable effort from serum samples PSP methodology is based on the microscopic analysis of individual nanoparticles from denatured whole blood samples. The resulting polymeric structures can provide information about cell functions and disorders.

Currently, PSP analysis has been performed in the form of a generalized, empirically proven analysis process. To fulfill the requirements of standardization and accreditation, the structures underlying Polyp Specific Polymer (PSP) analysis must be registered by image acquisition [6].

In view of the potential of such methodology in the diagnosis of colon cancer, we have evaluated the diagnostic potential of Polyp Specific Polymer analysis (PSP) in a prospective, double-blinded, controlled clinical study.

## Materials and methods

### Trial design

The double-blind and multi-centric study was designed as a prospective, open clinical diagnostic study in Germany. The deciding inclusion criterion was the participation in a screening for polyposis and with no known disease. Patients over 50 years of life were included according to the recommendations of BMG (Federal Ministry of Health). The study was conducted in parallel with a screening colonoscopy according to current screening guidelines [7].

From September 2011 to May 2012, 233 patients entered the study. 9 physicians recruited the patients and recorded clinical data (n = 233), performed colonoscopy and biopsy (n = 216; abbreviated in results as RS), and took blood samples (n = 233). Medical examinations were carried out on 233 patients. 45 patients dropped out during statistical analysis (RS and PSP-test not available (N = 3); RS not available (N = 14); RS: D + and PSP-test not available (N = 22); RS: D- and PSP-test not available (N = 6)). So the analysis set has an amount of 188.

The study was deemed a phase III trial according to the criteria of the German Society on Medical Informatics, Biometry, and Epidemiology (GMDS; http://www.gmds.de) allowing direct estimation not only of sensitivity and specificity, but also of predictive values. The primary objective of this clinical trial was to estimate the diagnostic accuracy (sensitivity and specificity) of PSP analysis for colon polyps using colonoscopy as a diagnostic accuracy standard. Secondary objectives of this trial included estimating positive and negative predictive values for colon polyps, investigating reliability, determining covariates influencing diagnostic accuracy, and obtaining absolute and relative frequencies of valid test results.

The study was registered with German authorities (*Deutsches Institut für Medizinische Dokumentation und Information*, DIMDI) under Number 00006854. The local ethics committee (*Ethikkomission Landesärztekammer Sachsen*) approved the study under Number EK-BR-55/10-1 and all participants signed the informed consent documentation. Nine medical doctors in private practice recruited patients and performed colonoscopy and biopsy. Biopsy specimens were analyzed in regional pathology centers. Blinding, de-blinding, and randomization were carried out at the Translational Centre for Regenerative Medicine (TRM) of the University of Leipzig. Statistical evaluations and data analysis were performed by ACOMED statistics (http://www.acomed-statistics.com).

The parallel colonoscopy could be performed on the day of blood sampling or later, whereby a maximum time difference of 2 weeks was allowed. The following parameters were evaluated during the screening visit: sex, name, date of birth, blood pressure, heart frequency. Inclusion and exclusion criteria are listed below.

Inclusion criteria:

• Informed consent provided.

• Patient was capable of providing an adequate health history.

• Age 50 years or older at the time of colonoscopy (colorectal screening guideline eligible).

• Accessible for blood withdrawal prior to the start of bowel preparation for colonoscopy.

• First large bowel endoscopy in patient lifetime.

Exclusion criteria:

• Terminal illness.

• Presence of severe psychiatric symptoms.

• Colonoscopy or another FOBT within the previous 2 years.

• Anorectal bleeding or hematochezia within the last 6 months for which the patient sought medical attention.

• Known iron deficiency anemia within the last 6 months for which the patient sought medical attention.

• Previous history of colorectal polyps or CRC.

• High risk for colorectal cancer (2 or more relatives with CRC; 1 or more relative(s) < 50 years with CRC; known HNPCC or FAP).

• Myeloproliferative disease.

• Lack of compliance.

The colonoscopy results combined with biopsy histological examination were used as the diagnostic accuracy standard (DAS) for the primary objective (i.e. detection of adenomatous polyps). The results were classified as below:

• Colonoscopy negative and histology negative (DAS negative).

• Colonoscopy positive and histology negative (DAS negative).

• Colonoscopy positive and histology positive, (DAS positive).

### Laboratory diagnostics

PSP methodology is based on the microscopic analysis of thermal denatured, resolved and crystallized whole blood samples.

The Polyp Specific Polymer (PSP) test has been used to analyze characteristic changes in whole blood samples after prior thermal decomposition and crystallization in the nanoparticle range [8]. Analysis of the results requires a quantitative and qualitative description of crystallization patterns and intensities similar to X-ray crystallography/structure analysis [9]. All steps in the procedure are accurately described using a validated Standard Operating Procedure (SOP). Evaluations were carried out by collaborators using currently existing clinical comparison standards. PSP analysis involves the processing of large amounts of data. The analysis was carried out visually on the basis of pattern matching as described in following sections.

### Polymer synthesis

1. The procedure is initiated with the thermal decomposition of the blood sample. For this purpose, the product is denatured by a standardized method to carbohydrate chains and peroxide compounds. During method validation, this was confirmed by scanning electron microscopy (SEM) with energy dispersive x-ray spectrometry (EDX) for particle characterization, using an automated electron beam to monitor the laboratory process. Electron microscopy provides not just visual information about the organic and inorganic submicron particles (size, shape, and morphology), but also chemical identification as verified by x-ray spectrometry [8].

2. The subsequent re-suspension of the collected particles facilitates re-polymerization and the crystallization into a solid form (i.e. based on the previously formed polymer structures) as monomers and crystals. The size of these monomers and crystals is recorded as an in-process control. Dynamic Light Scattering (using a Zeta-Size-Nano, Malvern) is used to measure particle size. This technique measures the diffusion of particles moving under Brownian motion, and converts this to size and a size distribution using the Stokes-Einstein relationship. Non-Invasive Back Scatter technology (NIBS) is incorporated to give the highest sensitivity simultaneously with the highest dynamic size and concentration range. The validated size of the particles should be between 60 and 600 nanometers.

3. Insoluble residues are removed by filtration.

4. The filtrated supernatant is spread on prepared slides for evaporation.

5. Following dehydration, the particles are precipitated in complexes forming polymers and crystal textures.

6. These structures are analyzed microscopically and documented digitally.

### Pattern acquisition

**Figure 1:**
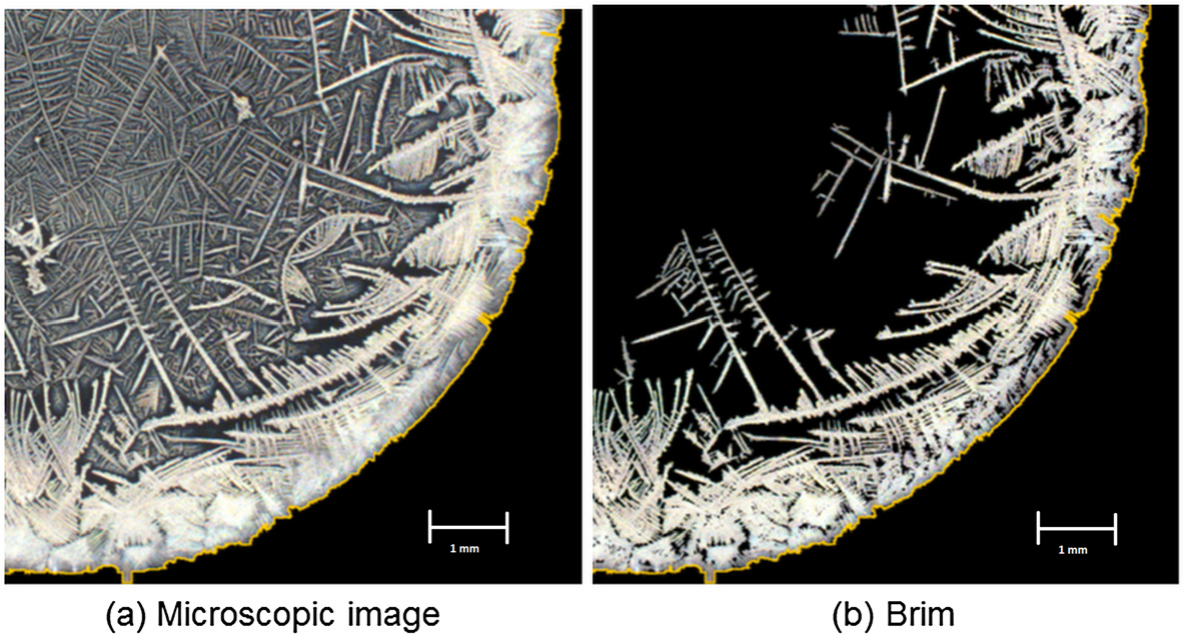
Example findings representing structures considered typical for polyps (original magnification 25x) (a) shows all connected structures while (b) shows the brim.

To support easier standardization and validation, patterns were acquired by a digital computer camera using imaging software and analyzed visually for target structures using the same software (Zeiss Imaging Solutions 4.8″; Zeiss, Oberkochen, Germany). The pattern indicating polyps was experientially described and documented. The truncated image shown in Figure 1 is representative of the structures used in established visual PSP analysis.

### Image analysis

Following microscopic image acquisition, textures and patterns as used in visual examination had to be recognized by image analysis. By using the LabImage platform (Kapelan Bio-Imaging, Leipzig, Germany), an algorithm was developed that allowed the traceable detection of PSP-relevant patterns.

## Results

### Recruitment of patients

From September 2011 to May 2012, 233 patients entered the study. 9 physicians recruited the patients and recorded clinical data (n = 233), performed colonoscopy and biopsy (n = 216), and took blood samples (n = 233). Medical examinations were carried out on 233 patients. 45 patients dropped out during statistical analysis (RS an PSP-test not available (N = 3); RS not available (N = 14); RS: D + and PSP-test not available (N = 22); RS: D- and PSP-test not available (N = 6)). So the analysis set has an amount of 188. The results are comprised of 17 (7.3%) cases with missing/unclear data, 165 (70.8%) cases with negative results, and 51 (21.9%) with positive results. Out of these patients, 33 had polyps confirmed by colonoscopy and histology.

### Laboratory analysis

PSP analysis could be carried out in 202 patients and revealed 114 patients with a negative result (48.9%) and 88 patients with a positive result (37.8%) with colon polyp specific structures identified, as shown in Table 1. Missing and unclear results were detected in 31 cases (13.3%).

**Table 1:** Cross-tabulation for reference standard (RS = target condition) and test result (PSP-test), absolute and relative frequencies

**PSP-Test**	**RS**				**All**	
	**D-**		**D+**			
	**N**	**%**	**N**	**%**	**N**	**%**
T-	99	62.3	8	27.6	107	56.9
T+	60	37.7	21	72.4	81	43.1
All	159	100.0	29	100.0	188	100.0
**Diagnostic accuracy (Sens, spec)**						
Sens:	72.4% (width of Cl: 52.8%.. 87.3%)					
Spec:	62.3% (width of Cl: 54.2%.. 69.8%)					
Missings	RS					
	D-		D+			
Total	159 + 6 = 165	29 + 22 = 51	188 + 28 = 216	Total	159 + 6 = 165	29 + 22 = 51
Prev:	23.6%					
**Diagnostic accuracy (predictive values acc. Bayes theorem)**						
PPV:	37.2% (width of Cl: 30.5%.. 44.5%)					
NPV:	88.0% (width of Cl: 80.0%. 93.0%)					
Unknown RS	17				17	
Total					233	

### Image acquisition

**Figure 2:**
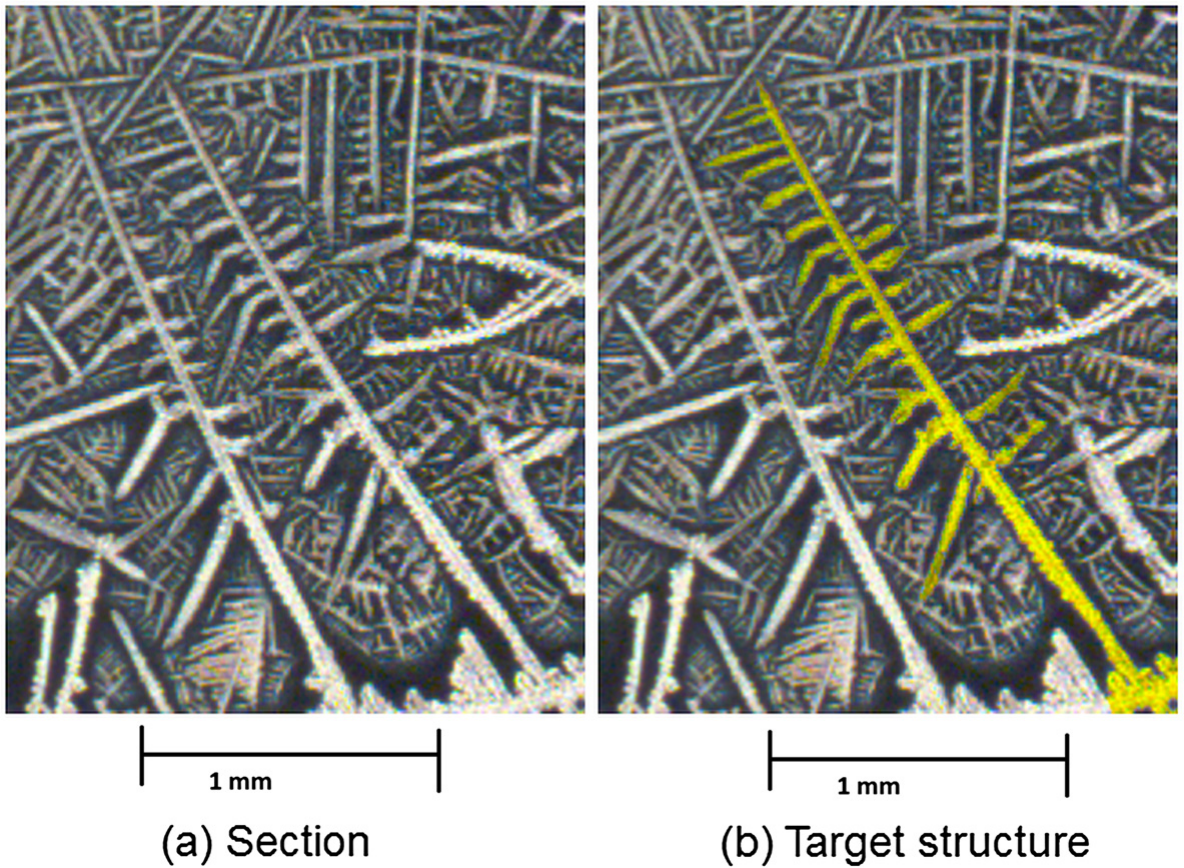
**Patterns indicating polyps. (a)** and **(b)** show examples of the pattern indicating that polyps can informally be described as one elongated stem with rather short branches or teeth connected to the stem which almost never branch out further (original magnification 100x).

The first criterion to identify a target structure is the connection to the crystal brim in the microscopic image. This criterion is necessary and structures not connected to the brim are not considered target structures even if they would otherwise fit the pattern criteria. Figure 1(a) shows all connected structures for the image while Figure 1(b) shows the brim. The pattern indicating polyps can informally be described as one elongated stem with rather short branches or teeth connected to the stem that rarely or never branch out further (Figure 2).

**Figure 3:**
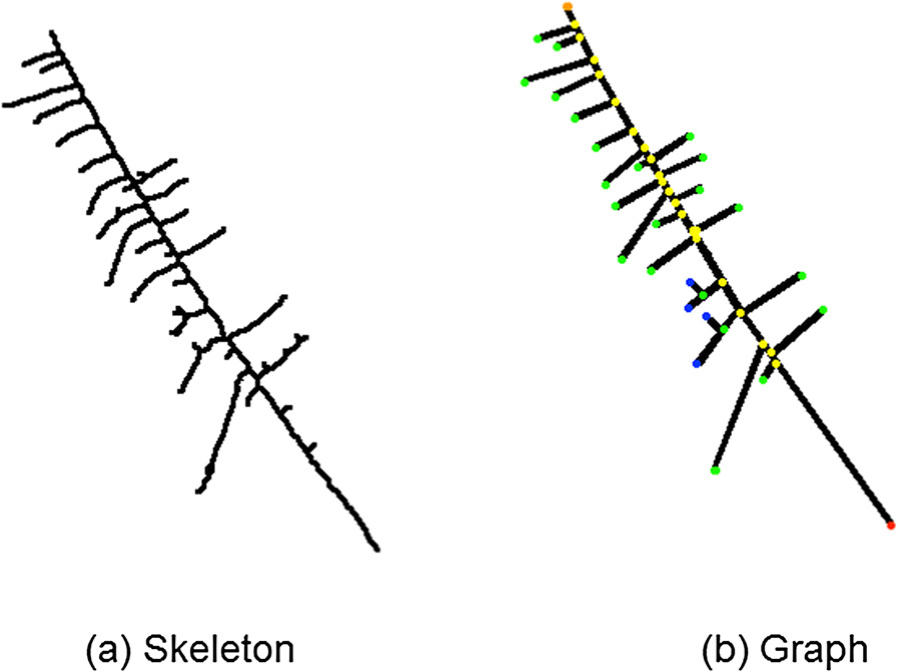
**Description of the crystal patterns as tree-graphs (a).** Vertices are colored in **(b)**, description of the colors in the text.

To formalize the pattern, we used graph theoretic descriptions [10]. We described the crystal patterns as tree-graphs (Figure 3). A graph *G = (V, E)* is an ordered pair, where V is the set of nodes (vertices, colored in Figure 3(b)) and E is the set of edges (black in Figure 3(b)) connecting elements of V. We have only considered graphs where *e(u,v)∈E→u ≠ v* (no loops) and multiple edges (different edges connecting the same nodes) do not exist.

A graph *T = (V, E)* is a tree, if it contains no cycles and is connected. A cycle is a path, where the first and the last node of the sequence are the same. Two nodes *u, v ∈ V* are connected, if a path in T exists. A path is a sequence of nodes *P = {v*_*1*_*,…v*_*n*_*}∈V* with *n ≥ 2* and for every pair *v*_*i*_*, v*_*i+1*_*;1 ≤ i < n - 1* there exists an edge *e{n*_*i*_*,n*_*i+1*_*}∈ E* connecting those nodes. A graph is connected, if any pair of distinct nodes in the graph is connected.

If we let *T = (V, E)* be the tree graph of a crystal pattern, as shown in Figure 3(b), to describe the target structure we need to define the root ρ of T, which is the node closest to the brim (red node in Figure 3(b)). We define the stem *S(V*_*s*_*, E*_*s*_*)* of T as the path from ρ to one *w ϵ V* where the length of the path is maximal *max*_*i*_*|P| = |ρ,…,v*_*i*_*|*. Note that there are at least two, maybe several nodes, w (depending on the tree topology), where the length of the path from the root is maximal. For our purpose, it does not matter, which w is chosen since if there are several possible stems, the topology cannot fulfill the criterion as mentioned below. In the example in Figure 3(b) the orange colored node at the tip of the tree is chosen as w. We now assign a distance labeling on the edges, where the edges of the stem are labeled with 0, edges adjacent to the stem with 1, edges adjacent to those with 2 and so on. Formally: *d(e) = 0* if *ϵ E*_*s*_ , also *d*(*e*{*u, v*}) = 1 if *e*  ∉ *E*_*s*_, but either *u ∈ V*_*s*_ or *v ∈ V*_*s*_ and *d (e) = min (d(f{x, y}) + 1* where *f ∈ E* and *x = u* or *x = v* or *y = u* or *y = v*.

In Figure 3(b) the trunk is comprised of the red, orange, and yellow nodes as well as all edges connecting them. Therefore, an edge, e, in Figure 3(b) connecting yellow and green nodes is labeled with d(e) = 1 and an edge, f, connecting green and blue nodes is labeled with d(f) = 2. The target structures are now characterized by having a significant number of edges where (e) = 1, which are also rather short if compared to the stem, while there are few or no edges with d(f) = 2 and no edges with d(e) ≥ 3 present. A binary test yields “colon polyp is present” vs “colon polyp is not present” as results. In addition, the result “not assessable” may occur.

### Statistic description of PSP test characteristics

Results from 188 patients were included to establish a 2x2 table comparing the test results of the PSP analysis with the results of suspicious cells from histological examinations. As demonstrated in Table 1, 99 cases (62.3%) were presented for type 1 (negative/negative), 8 cases (27.6%) for type 2 (positive/negative), and 60 cases (37.7%) for type 3 (negative/positive). For type 4 (positive/positive) 21 cases (72.4%) were presented. Taken together, a sensitivity of 72.4% (95%-CI: 52.8%-87.3%) and a specificity of 62.3% (95%-CI: 54.2%-69.8%) could be proven.

As predictive values, a PPV of 37.2% (95%-CI: 30.5% – 44.5%) and a NPV of 88.0% (95%-CI: 80.0% - 93.0%) were found. Hereby, a prevalence of 23.6% was assumed, which is yielded when cases with known RS but missing test results are taken into account additionally (see Table 1, lower part). This prevalence is in coincidence with known prevalence of polyps in German population. The polymer structures can be represented qualitatively. Similar to other crystallographic methods, unique patterns are sought that occur specifically in relation with the pathology. Rather than being measured as a quantitative value, the patterns are looked at for qualitative changes in both the shape and size of the crystals produced. Therefore, these are the only two options available for the evaluation.

Those subjects with adenoma-carcinoma activity in the colon exhibit differentially expressed genes in the tissue and exhibit changes in the concentration of various substances in the whole blood which result from the gene mutation typical for adenoma [11]. These cause a change in the shape of the polymers, as described in the Image Analysis section. Logically, subjects with such structures exhibit a positive result (i.e. a serious suspicion of adenoma polyps in the colon) while subjects without such structures receive a negative result.

To check the operator dependency, the results of both readers (in our case collaborators who evaluate the structures) were compared. The results show that in 129 cases, reader 1 and reader 2 evaluated the structures with “negative”, in 86 cases with “positive” while in only 14 (6.1%) of the cases did the readers give different results. In 93.9% of the cases, both readers reached the same conclusion. These results strongly suggest that the Polyp Specific Polymer analysis is operator-independent.

## Discussion

This study was undertaken to evaluate the diagnostic accuracy of a new non-invasive diagnostic laboratory methodology using routinely taken blood samples and PSP to detect adenoma polyps. In patients undergoing colonoscopy screening and histology examination, a sensitivity of 72.4% and a specificity of 62.3% was proven. In Western societies, malignant diseases are ranked as the second leading cause of death with only cardiovascular diseases ranked higher. Colorectal cancer (CRC) is the third leading disease with an increasing incidence. The detection of precursors at early stages of the disease is crucial for reducing the CRC mortality rate. The incidence of individuals choosing to undergo voluntarily the preventive examination currently offered for everybody older than 55 years of age in Germany is very low. In fact, it is as low as the sensitivity of the entirely non-invasive risk detection method using stool tests. Despite the increase in public acceptance of FOB testing, there is no expected increase in its sensitivity for detecting polyps.

### Fecal occult blood testing

Fecal occult blood (FOB) testing is a simple, non-invasive test that can be carried out by most primary care physicians. There are several studies suggesting that annual FOB testing, especially if combined with sigmoidoscopy may decrease the mortality of colorectal cancer. In recent years there has been some controversy regarding the effectiveness of the different types of FOB tests since in general they are known to generate a high number of false positives. In CRC screening trials, between 0.8% and 15% of participants tested had a positive FOB test result while 55–65% of participants with a positive FOB test result had no colorectal cancer or adenoma [12-15].

Original FOB tests, also called guaiac-based or gFOBT detect the heme derived from blood in the stool. In 2001, a new class of FOB tests called Fecal Immunochemical Tests (FIT) were introduced which detect globin in feces rather than heme. By detecting globin the tests are both more sensitive and specific for lower levels of gastrointestinal bleeding. Although these superior FIT tests are now recommended in place of the traditional annual standard guaiac FOBT, the older FOB tests are still commonly used [16,17]. A comparative evaluation of immunochemical fecal occult blood tests for colorectal adenoma detection is presented in Table 2.

**Table 2:** **Comparative evaluation of immunochemical fecal occult blood tests for colorectal adenoma detection**[17]

	**Hemoccult**	**Immunological tests for:**					
		**Hemoglobin**					**Hemoglobin/haptoglobin complex**
		**Bionexia FOB plus**	**Prevent IDCC**	**Immo-care C**	**FOB advanced**	**Quick vue iFOB**	**Bionexia Hb/Hp complex**
Sensitivity %	5.4	35.8	29.6	11.4	18	45.2	58
Specificity %	95.9	81.9	81.8	96.7	92.9	70.2	58.8
Scatoscopy	X	X	X	X	X	X	X
Blood analysis							

### Virtual colonoscopy

Virtual colonoscopy is an exam used to detect changes or abnormalities in the large intestine (colon) and rectum. During virtual colonoscopy, computerized tomography (CT) is used to produce hundreds of cross-sectional images of the abdominal organs. The images are combined and digitally manipulated to provide a detailed view of the inside of the colon and rectum. Unlike traditional colonoscopy, virtual colonoscopy does not require sedation or the insertion of a scope into the colon. The high cost of CT exams as a screening tool remains a negative factor.

### Colonoscopy

For those patients who have tested positive for blood in the feces (positive FOB or FIT test) colonoscopy is mandatory. Colonoscopy is now accepted as the most accurate method of detecting colonic polyps and also allows simultaneous removal of most lesions. Resected polyps are typically examined pathologically and categorized as benign adenoma (tubular, tubulovillous, or villous), carcinoma in situ, or invasive cancer. Appropriate follow-up testing, usually colonoscopy, is mandatory for patients with positive results from their first examination. Since most clinically significant colon polyps are located distal to the splenic flexure, flexible sigmoidoscopy may be a reasonable alternative to colonoscopy. However, lesions in the right colon may go undetected and those patients found to have a polyp on flexible sigmoidoscopy will consequently also require a full colonoscopy, therefore being subject to both tests [18-20].

### PSP testing

PSP testing is a laboratory method based on thermal denaturation of venous blood, followed by resuspension, drying, and crystallographic read-out. Crystallographic methods for the characterization of biological materials in non-homogeneous, very complex bodily fluids have been used in diagnostic investigation for a long time. Cerebrospinal fluid, saliva, and cervical mucus have been successfully treated crystallographically and subjected to various types of diagnosis [21-25]. Currently developed procedures of PSP analysis require qualitative analysis of the microscopic textures by a laboratory physician (reader).

The patterns are associated with different pathological processes. Their composition has been analyzed by electron microscopy and radiological measurements (SEM-EDX: Scanning electron microscopy (SEM) with energy dispersive x-ray spectrometry (EDX) for particle characterization using an automated electron beam) can be used to monitor the production process. Electron microscopy provides not just visual information about the organic and inorganic sub-micron particles (size, shape, and morphology), but also chemical identification based on the X-ray energy lines, empirical evaluations, and biochemical methods. The complex electron microscopic and radiological techniques are necessary only during the research phase. Because the dehydrated end-products’ optical structures are uniquely recognizable, optical light microscopy can be used, which requires less sample preparation.

The evaluation of the results arising from PSP analysis involves processing large amounts of data, which has so far been carried out visually on the basis of pattern matching. However, to facilitate a wider adoption of PSP and integration into daily practice it will be necessary to validate the test through further laboratory testing [6]. Detecting relationships between disease patterns and outcomes in polymer analysis is a major technological challenge, because the analyzed structures can vary greatly in shape, size, composition, and structure. Many analytical methods that measure these textures therefore only take an average over a large collection of them and do not measure individual properties.

One of the unique characteristics of INDAGO’s methodology is the ability to ensure stability in the pattern, shape, size, composition, and structure throughout the whole blood denaturation step, which had previously been considered impossible. As a result, for the first time it has been possible to develop a basic understanding of the relationship between the analytical results and the disease. This is time consuming and to some extent subjective being strongly dependent on personal expertise. Although INDAGO is in the process of automating the evaluation process, for the purpose of this trial, two independent laboratory readers evaluated the results under masked conditions. For the evaluation of diagnostic accuracy, the final result was derived according to the following rules: If the two readers agreed in a positive or negative test result, this test result was used as final test result. If the results of the two readers were different (e.g. positive for reader 1 and “not assessable” for reader 2), the final result was determined by an adjudication meeting of the two readers. For the meeting, the aliquots (but not the samples) were un-blinded by the TRM.

Therefore, approximately 220 tests were used for the calculation of the primary and secondary endpoints. In this study, PSP analysis, in relation to full colonoscopy as the reference with its own sensitivity of around 80%, offered a low-risk sensitivity of 72.4% at a specificity of 62.8%. We believe that this represents for the first time the possibility to effectively identify the highest-risk candidates for endoscopy. This improved screening advances the ultimate aim of decreasing the incidence or mortality of colorectal cancer in the selected population. The high amount of false-positive PSP results may be due to the relatively low sensitivity of colonoscopy (76% for small polyps), which is considered the “Gold-Standard” detection method. Significantly, PSP analysis has the potential to conserve socio-economic resources by identifying and subjecting more “right” and fewer “wrong” patients to endoscopy and transferring them to curative prevention via polypectomy or CRC early detection. By combining the best-established, low-risk screening elements with a blood test-based diagnostic test such as PSP that has good acceptance, sensitivity, and specificity, an increase in the reliable estimation of a patient’s risk for CRC before colonoscopy is expected. In the intermediate-term it is anticipated that public acceptance of colonoscopy will improve which will be a major gain for preventative health care.

Based on the current schemes for follow-up or preventive controls, the introduction of a PSP blood test would lead to better and more individualized patient care. The test detects colon adenoma/carcinoma-specific sequences of polymers individually, in any chosen time interval. It is noteworthy that no adenocarcinoma case was missed through PSP testing. In addition, sessile polyps were detected with a good sensitivity. Using PSP analysis, patient risk is extremely low since blood sampling is generally considered, at least under normal circumstances, a “low risk” procedure.

According to the Robert Koch Institute, the average age at which cancer is contracted is 73 (women) and 68 years (men) [1]. Every year, in Germany more than 70,000 men and women develop intestinal cancer and 30,000 die from it. In October 2002, the legislature amended the provisions of the law governing the early detection of intestinal cancer. Between 50 and 55 years of age one annual stool test for concealed blood (FOBT) is offered.

During their 56th year of life all statutory insurance members are entitled to their first colonoscopy.

Due to fear, shame, uncertainty, repression, and lack of knowledge this free offer is only utilized with hesitation. Additionally occasional long waiting times augment such personal reservations people may have about the intestinal cleansing. Therefore, it is necessary to take steps in order to increase the early detection rate.

As a non-invasive method, the PSP-blood test is patient-friendly and thus makes an important contribution to a wider acceptance of preventive measures and the participation in regular preventive check-ups for intestinal cancer.

Equally important is that the PSP test detects high-risk polyps, which left alone will directly lead to cancer. Removing them is a very effective way to prevent colon cancer from developing.

Colon polyps have been demonstrated through autopsy studies to occur in more than 30% of people over the age of 60 and in recent studies, large-scale screening (more than 250,000) of asymptomatic German patients over 55 demonstrated that 20% were found to have colonic neoplasia s. This same study also showed that 7% were diagnosed with advanced adenoma and invasive cancer was demonstrated in 0.8% of the population [26].

Colon cancer represents one of the most important causes of death in the elderly. Polyps are considered pre-cancerous and causal for this disease. Despite a substantial number of false-negative findings and generally poor acceptance by patients, screening is still performed by colonoscopy. In contrast, the relative non-invasiveness of PSP-analysis, a novel blood based assay requiring only simple blood collection, accords better patient acceptance as well as providing a sensitive and selective means for the early detection of colon polyps, particularly those high-risk polyps which if left in situ can lead directly to cancer.

## Competing interests

We have read the journal’s policy and have the following conflicts to declare: Luis I. Gomez Fernandez is CEO of INDAGO GmbH. Markus Franz has been employed by the Kapelan Bio-Imaging GmbH.

## Authors’ contributions

MF carried out the image analysis and drafted the manuscript. MS carried out colonoscopy. IH collected data and participated in the statistical analysis. SR participated in the design of the study and carried out colonoscopy. LG conceived of the study, participated in its design and coordination, and drafted the manuscript. All authors read and approved the final manuscript.
